# Motivational Interviewing Conversational Agent for Parents as Proxies for Their Children in Healthy Eating: Development and User Testing

**DOI:** 10.2196/38908

**Published:** 2022-10-07

**Authors:** Diva Smriti, Tsui-Sui Annie Kao, Rahil Rathod, Ji Youn Shin, Wei Peng, Jake Williams, Munif Ishad Mujib, Meghan Colosimo, Jina Huh-Yoo

**Affiliations:** 1 College of Computing and Informatics Drexel University Philadelphia, PA United States; 2 College of Nursing Michigan State University East Lansing, MI United States; 3 Tata Consultancy Services Edison, NJ United States; 4 College of Design University of Minnesota Minneapolis, MN United States; 5 College of Communication Arts and Sciences Michigan State University East Lansing, MI United States; 6 Vivante Health Houston, TX United States

**Keywords:** conversational agents, voice user interface, voice agents, proxy, motivational interviewing, parents, healthy eating

## Abstract

**Background:**

Increased adoption of off-the-shelf conversational agents (CAs) brings opportunities to integrate therapeutic interventions. Motivational Interviewing (MI) can then be integrated with CAs for cost-effective access to it. MI can be especially beneficial for parents who often have low motivation because of limited time and resources to eat healthy together with their children.

**Objective:**

We developed a Motivational Interviewing Conversational Agent (MICA) to improve healthy eating in parents who serve as a proxy for health behavior change in their children. Proxy relationships involve a person serving as a catalyst for behavior change in another person. Parents, serving as proxies, can bring about behavior change in their children.

**Methods:**

We conducted user test sessions of the MICA prototype to understand the perceived acceptability and usefulness of the MICA prototype by parents. A total of 24 parents of young children participated in 2 user test sessions with MICA, approximately 2 weeks apart. After parents’ interaction with the MICA prototype in each user test session, we used qualitative interviews to understand parents’ perceptions and suggestions for improvements in MICA.

**Results:**

Findings showed participants’ perceived usefulness of MICAs for helping them self-reflect and motivating them to adopt healthier eating habits together with their children. Participants further suggested various ways in which MICA can help them safely manage their children’s eating behaviors and provide customized support for their proxy needs and goals.

**Conclusions:**

We have discussed how the user experience of CAs can be improved to uniquely offer support to parents who serve as proxies in changing the behavior of their children. We have concluded with implications for a larger context of designing MI-based CAs for supporting proxy relationships for health behavior change.

## Introduction

### Motivation

Off-the-shelf conversational agents (CAs) such as Google Home, Amazon Alexa, and Echo are becoming increasingly popular for managing health in everyday settings. More than 50 million purchases of Amazon Echo have been made in the United States alone [1]. CAs have also been shown to help in improving parenting practices. CAs can help set up children’s pre-established healthy diet–based plans as well as help track children’s food consumption [2]. Despite the benefits of CAs in supporting a family’s healthy eating habits, studies have also highlighted limitations that still need to be addressed. A study on the perception of mealtime technologies showed that parents did not like the idea of technology telling them or their children what to do and wanted more control over their family’s food decisions [3]. Furthermore, parents believed the use of CAs would increase the dependence of their families on technology [3]. These findings point to the need for technologies such as CAs to support parental control and involvement in the meal decisions of their children.

Approximately 40% of US children’s daily caloric intake consists of high-fat foods or sugary drinks, raising concerns about linked chronic illnesses due to unhealthy eating [4]. Parents perform a critical role in shaping children’s eating habits and are often the decision makers for the family meal [5-7], which poses a long-term health effect as adults. Parents take on the responsibility and accountability of bringing about a health behavior change in their children that they care for. This form of relationship, which we call a *proxy relationship*, is a socially mediated relationship that involves giving a person—the proxy—who supports the target individual of behavior change the power to achieve the target individual’s goals [8,9]. Studies on CAs for family use often focus on direct interactions with the target individual, treating behavior change as an individual problem. However, in the case of children who may not have access to or know how to correctly use CAs, parents can interact with CAs and can serve as a proxy for behavior change in their children.

For parents serving as proxies, factors such as lack of motivation, time, and work often get in the way of parents practicing healthy eating habits together with their children [10-12]. The Motivational Interviewing (MI) technique has emerged as an effective counseling method that helps individuals discover motivations and strategies for personalized behavior change [13], especially for diet modification [14-16]. MI emphasizes individual autonomy and helps individuals self-form personalized solutions that might work for their physical, social, and economic constraints [13,17]. Technology-based MI approaches deliver adaptations of MI using technology and various types of media including CAs [18]. These technology-based approaches have been shown to extend the MI intervention beyond what a therapist could offer face-to-face and provide cost-effective access to therapeutic services to underserved clients, such as rural populations [19]. However, current technology-based MIs are focused on direct interactions with the target individual of behavior change. This approach might not be as useful for those target individuals with low literacy, agency, or little motivation who may not or cannot interact with technology directly. This limitation of MI-based technologies is especially true for young children, who are often dependent on their parents to make decisions on their behalf. Behavior change in this context is a social problem; however, existing MI-based CAs often overlook this social context of behavior change. More work is required to design CAs for addressing behavior change problems that require more than one individual to work together (eg, parent and child), where one individual is dependent on the other to make decisions on their behalf, for instance.

In this study, we investigated how MI-based CAs can help address the gap in supporting health behavior change facilitated by proxy relationships. To explore the requirements for this design opportunity, we developed a working prototype of an MI-based CA called Motivational Interviewing Conversational Agent (MICA). The MICA prototype incorporates automated MI to help parents of young children adopt healthy eating practices. As parents are motivated to eat healthy using MICA, they will be better supported to inculcate similar healthy habits in their children. The end goal of MICA is to support parents serving as proxies for their children in healthy eating through cost-effective access to MI. Our objectives were to understand (1) how parents perceive MICA to best deliver its interventions to help them serve as proxies for health behavior change for their children and (2) how parents experience a CA, such as MICA, for behavior change to understand how families use in-home CAs for managing everyday practices. Our study is a starting point toward MI-based CAs supporting proxy relationships wherein the proxy individual facilitates a behavior change in the target individual.

### Background

#### Parents as Proxies for Behavior Change in Their Children

A proxy relationship is a socially mediated relationship that involves a person (proxy) having the power to achieve another person’s goals [8,9]. Proxies often help individuals rely on them to accomplish goals when the individuals (1) do not have the adequate means or resources to reach their goals, (2) have more trust in the proxy than themselves to lead them toward behavior change, and (3) do not want to be accountable for reaching their goals. This proxy relationship allows target individuals of behavior change to make better decisions, invest appropriate knowledge and time, and be accountable for managing their health. Target individuals of behavior change may also lack the ability to make informed choices during their behavior change process because of cognitive or physical disability or lower technical and health literacy. For instance, parents serving as a proxy and helping their children in health behavior change can be especially beneficial for the child where the child may not be able to make informed decisions. Parents also play a critical role in how children establish their long-term eating habits from a young age [7,20,21]. Poor diet during childhood leads to obesity, which is also highly linked with chronic illnesses, including diabetes and cardiovascular diseases [22].

Despite the critical role parents play, they often have the extra burden of not getting enough time and support for themselves [23]. Supporting parents can help reduce their burden and lead to extended periods of parenting. Researchers have identified challenges for parents in the form of a negative psychological response to the obligations of being a parent [24]. Such a response can negatively affect the motivation and self-esteem of parents to adopt healthy behaviors as a family [25]. A scoping review of health-related parenting showed that the areas where parents require support are accessing complex information, guiding through decision-making processes, or self-monitoring with appropriate feedback [26]. Parenting programs help parents in mitigating these challenges and motivate parents to provide better support to their children. However, parents face barriers to participating in parenting programs that are highly correlated with their socioeconomic status, material hardship, and resource limitations (eg, transportation issues and childcare needs) [27-29]. Such cost-related barriers can limit parents’ access to and use of the available resources.

Emerging technologies, such as CAs, have the potential of providing support to parents in a cost-effective manner, suited to the needs of parents and their children [30-32]. However, in the case of parents wanting to eat healthier together with their children, direct interaction of the technology with the children may not be possible or practical because of the constraints around technology accessibility, literacy, or accountability. More work is required to understand how technology can work with parents to help them serve as a proxy and change the health behavior of their children.

#### In-home CA Use for Parenting

For the last few years, an increasing number of studies have looked at how in-home CAs can support a wide array of tasks for improving family practices. As off-the-shelf CAs became available on personal devices, people can adopt them at reasonable costs compared with the early forms of laboratory-based CAs. CAs can explain complex concepts using simple communication techniques without the technical literacy constraints that web-based patient portals generally require [33]. CAs also help users verbalize their goals for behavior change [34]. CAs offer the potential of customization to fit better the needs and goals of individuals with low health and technical literacy [30,35].

Several studies showed that in-home CAs can augment family practices by helping parents perform and regulate day-to-day tasks [2,36,37]. For instance, field studies by Beneteau et al [36,37] showed that in-home CAs facilitated collaborative learning experiences in families. Researchers have also conducted content analysis and collected use data of households using in-home CAs. The results showed how agents are integrated into the family’s daily routines and used for varied purposes by different family members [38-41]. However, CAs lacked contextual knowledge of the households they were placed in and the activities that happened around them, often leading to privacy concerns [41]. In addition, CAs are not best equipped for children because parents have to be heavily involved, at least in the initial stage of CA use. For example, parents have to teach children how to effectively use commands and communication styles to use the CA better and safely [2,42,43].

A few recent studies examined how CAs can support healthy eating in parent-child dyads. For example, Garg and Sengupta [2] discussed that parents could use in-home CAs to track children’s food consumption and nudge children to eat healthier according to their rules. Jo et al [44] conducted a field trial of a sensor-based speech recognition system named MAMAS for promoting healthy eating behaviors in parents and children. They found that the use of MAMAS helped promote autonomy in children’s eating behaviors and positively affected family eating practices. These studies showed the benefits of family-based CAs in establishing household rules and values and the potential CAs have in improving children’s learning and eating habits. However, Chen et al [3] found that parents were skeptical about using CAs at mealtime for their children, as they perceived it to be distracting and intrusive. Chen et al [3] highlighted the importance of designing CAs in partnership with parents to account for their needs and trust. These studies did not focus on motivating behavior change because the CAs currently available in the market do not support goal-specific or context-specific conversations. In addition, these studies focus on the direct interaction of the CA with the target audience for behavior change; that is, either the parents or the children. However, in the case where the target individuals of behavior change are constrained by technology accessibility, literacy, or accountability, such as children, direct interaction of the CA with the target individuals of behavior change may not be possible or practical. More work is required to understand how CAs can help with parent-child proxy relationships.

#### Healthy Eating Through MI in Parenting Context

Among many factors that influence children’s eating patterns, parents’ feeding styles [45] and modeling parents’ preferences, intake, and acceptance of food play a significant role [46]. Accordingly, involving parents as core participants in interventions improves children’s healthy eating habits [22]. However, because of the complexities with parenting [12], lack of time [10], and maternal stress [11], even with parents’ desire to eat healthier as a family, motivation and confidence to eat healthier can be hindered [47].

MI, as a psychological therapeutic approach, provides a solution to this challenge by emphasizing individual motivation and autonomy. MI helps individuals self-form personalized solutions that might work for their physical, social, and economic constraints [13,17]. The MI technique has emerged as an effective counseling model for healthy eating and diet modification [15]. MI is a person-centered approach to help clients resolve barriers to motivation, reduce resistance, and foster commitment to lifestyle changes in modifying healthy behavior [16], especially when the change requires resolving various personal complexities [14]. In supporting effective long-term health behavior changes such as healthy eating, modified interventions such as MI are required that meet the needs of each individual’s behavior change stage at the time [48]. The transtheoretical model (TTM) of behavior change is an integrative theory of therapy that assesses individuals’ readiness and confidence to make changes to new healthier behavior and provides strategies accordingly [49]. The TTM has been instrumental in developing the MI technique, as the intrinsic motivation required to move through the different behavior change stages of the TTM is gained from the MI technique [50]. The TTM has been applied to a wide array of health and wellness contexts [51] with the MI technique to guide behavior change intervention design in the context of eating habits, such as women’s binge eating [52], eating disorders [53], and vegetable and fruit consumption [48].

Several studies investigated applying MI as part of the TTM in the context of parent-child dyads to examine the factors that influence parental readiness to facilitate their children’s weight management, including diet and physical activities. For example, Rhee et al [54] showed that health care providers’ suggestions based on MI or behavior change strategies facilitated parental readiness for children’s dietary behaviors. Another study also aimed to identify characteristics associated with parental confidence to change their family’s weight management behaviors, including eating and physical activities [55]. The results emphasized the importance of providing appropriate, targeted counseling for parents from the early stages, as overweight-related behaviors of children begin early in childhood. Similarly, in their focus group study, Bolling et al [56] discussed parents’ perspectives on effective approaches to managing their children’s weight that health care providers could make use of in their counseling. The results indicated that parents desired earlier, direct, and explicit interventions and suggestions. Studies suggest that earlier interventions involving early, direct counseling were positively associated with parents’ readiness and confidence to change their children’s dietary and weight management behaviors. In this regard, it becomes beneficial to introduce MI in the earlier stages of the parent-child dyad’s eating behavior. MICA incorporates MI starting from the first session to account for an early intervention incorporating strategies that parents come up with themselves to align with their goals. MI thus provides a way for CAs to take into account the goals and needs of parents supporting their children in the conversations for improving the TTM. As MI brings numerous benefits for parents supporting their children in behavior change, it becomes essential to understand how MI can help parents serve as proxies for their children.

Although studies highlighted the usefulness of in-home CAs in the parenting context, they mainly investigated the effectiveness of functions implemented in commercial products. The design requirements of CAs that support particular health behaviors for parent-child dyads, wherein the parents serve as proxies for their children, were not actively discussed. As families use in-home CAs for managing everyday practices, it is imperative to understand how parents experience a CA for behavior change. Furthermore, it is vital to understand how parents perceive it can best deliver its interventions to help them serve as proxies for health behavior change for their children.

## Methods

### Overview

We developed a working prototype of a MICA and conducted a user test remotely in a laboratory setting using semistructured interviews. We conducted 44 user test sessions of the MICA prototype remotely in a laboratory setting for 2 weeks with 24 parents of young children interested in healthy eating with their families. We used semistructured interviews to understand parent participants’ acceptance of using the MICA prototype and design opportunities to improve the MICA prototype. The goals of this study were to (1) understand parents’ perceived usefulness of the system in helping them change their own eating behaviors that can influence their children’s eating habits, (2) envision the ways in which MICA can help parents support their children to eat healthier, and (3) formatively evaluate how to improve the experience of using MICA to better support parents as proxies.

### MI-Based Conversation Script of MICA

**Figure 1:**
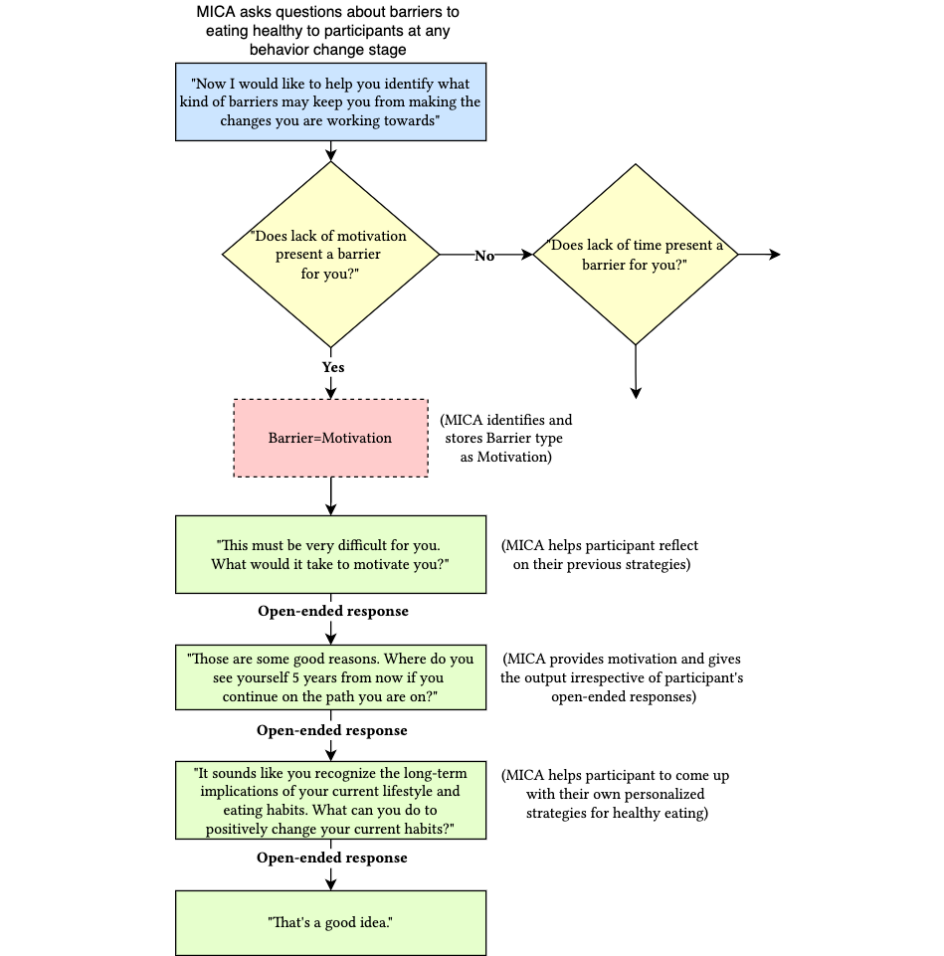
An excerpt from the Motivational Interviewing Conversational Agent’s (MICA) Motivational Interviewing (MI)–based conversation script. The logic tree structure of the script shows MICA’s responses to participants’ inputs of yes, no, and open-ended responses.

The current conversation script that MICA uses to converse with parents is based on MI scripts that human MI therapists follow. The MI script follows a logic tree structure (Figure 1). The MI script branches out into different subtopic questions based on the response of the participant.

An MI expert collaborator developed the conversation script to move parents from the preparation stage to the later action or maintenance stages outlined in the TTM [57-59]. The MI technique provides the conversational framework to achieve the goals of the TTM and drive conversations with MICA according to the needs and goals of the parents for eating healthy, thus giving parents autonomy over their decisions [13,17]. MICA uses the script to help parents serving as proxies for their children to become motivated to adopt healthier eating habits themselves. Parents can then inculcate the same habits in their children. More specifically, the script included six commonly identified eating problems (overeating, sweetened beverages, dining out, eating fast food, stressful eating, and out-of-control eating) for parent participants to identify and self-reflect based on the existing literature [60-65]. After problem identification (agenda setting), MICA guides participants to self-appraise their perceived importance and confidence in behavior change. MICA helps participants explore previous experiences in eating behavioral changes (both strengths and barriers) by asking various questions to provide opportunities to develop strategies to overcome the identified problem. Finally, MICA prompts participants to set achievable short-term goals for the upcoming weeks. MICA also discusses long-term goals to keep participants on track toward behavior change. The same conversation script, with minor revisions, was used by MICA in both sessions to see if the experience of using MICA changes between sessions 1 and 2. For CAs to be effective for health behavior changes, continuous interaction of parents with CAs is required over time [66]. Studies investigating the role of CAs in health behavior changes often look at the single-time use of CAs. To address this gap, we conducted 2 sessions with an interval of 2 weeks to examine the participants’ retention and possible changing perspectives about the system. The MI script was tweaked in the second session to include questions that follow-up on the progress with goals that the participants had set for themselves in the previous session. The conversation script also consisted of many open-ended questions beginning with *why* and *how* to help participants reflect on their habits. A part of the MI script built by our MI expert to show how MICA interacts with participants is as follows (from a list of challenges MICA presented, a participant identifies that eating out is the biggest challenge for eating healthy):

- MICA: “Thank you for sharing. It can definitely be hard to overcome a concern like this. On a scale of 0 to 10, how confident are you in making the change to eating out too much?”
- Participant: “5.”
- MICA: “It sounds like you are uncertain of your ability to change. Why not a higher number?”
- Participant: “I think time is always a concern. We don’t have time to go grocery shopping and cook every day.”
- MICA: “Thank you for sharing. What would need to happen for you to move to a higher number?”
- Participant: “I think I can at least try [cooking] once a week.”
- MICA: “That sounds like a plan. Any other plans you can think of?”
- Participant: “I think I can start planning the whole week and find time to go weekly grocery shopping.”
- MICA: “I would love to talk about how we can address this further.”

### The MICA Prototype

**Figure 2:**
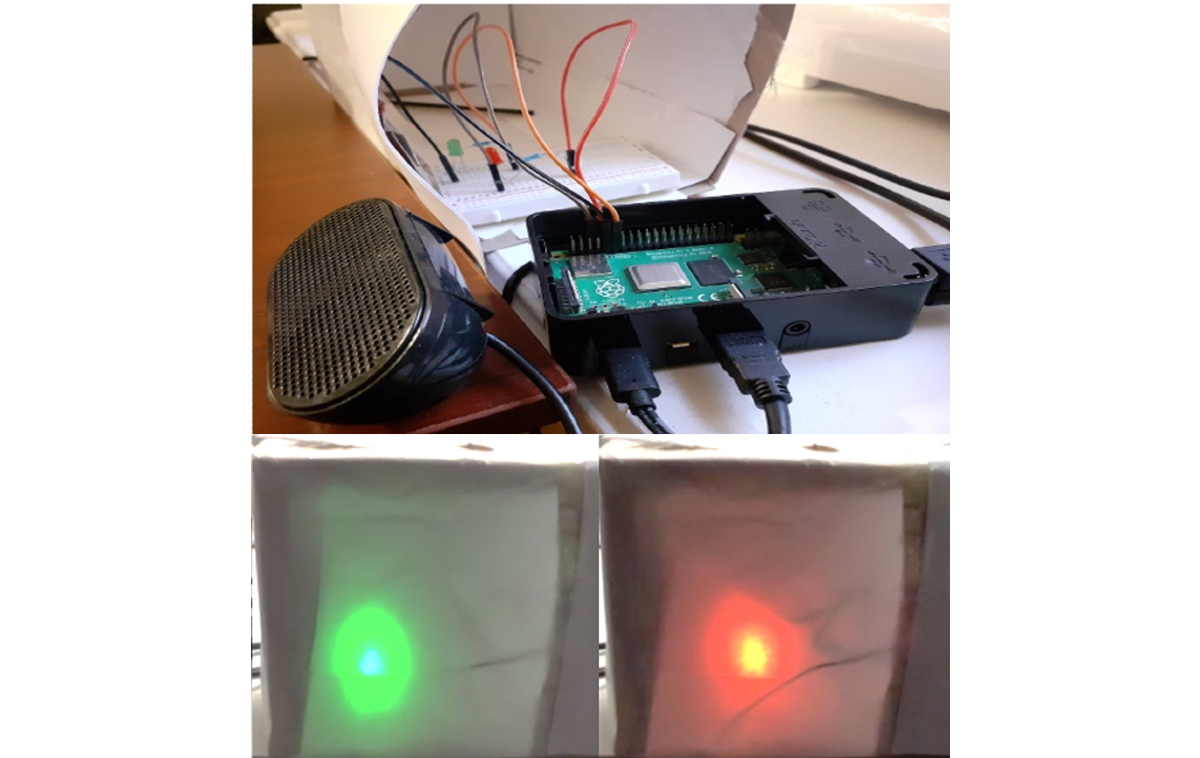
The top image shows the cross-section of the Motivational Interviewing Conversational Agent (MICA) prototype containing the Raspberry Pi, the breadboard with the LED lights and wires, and the speaker. The images on the bottom show the setup of MICA working prototype from the participant’s point of view used for the videoconferencing-based user study. The LED lights generate green and red lights. The green light indicates that MICA is ready to hear the participant speaking, and the red light is for when MICA is speaking to the participant.

The current MICA prototype runs on a Raspberry Pi platform, a computing kit manufactured by the Raspberry Pi Foundation to build computing technologies [67]. We used Python [68] to program MICA’s conversation script in a JSON [69] format. Instead of relying on a third-party company’s application programming interface, for example, Google Dialogflow [70] and Amazon Alexa [71], we built an in-house prototype to handle all data locally protected within the Drexel University network. The MICA prototype runs without any connection to the internet or engagement of third-party software that collects participants’ input. We decided to test the limits of how far the agent can perform without cloud-based high-performance computing to accommodate the privacy concerns of potential future parent participants and those in underserved settings with limited internet access. Figure 2 shows the MICA prototype’s cross-section and setup as shown to the participants.

### Data Collection

**Figure 3:**
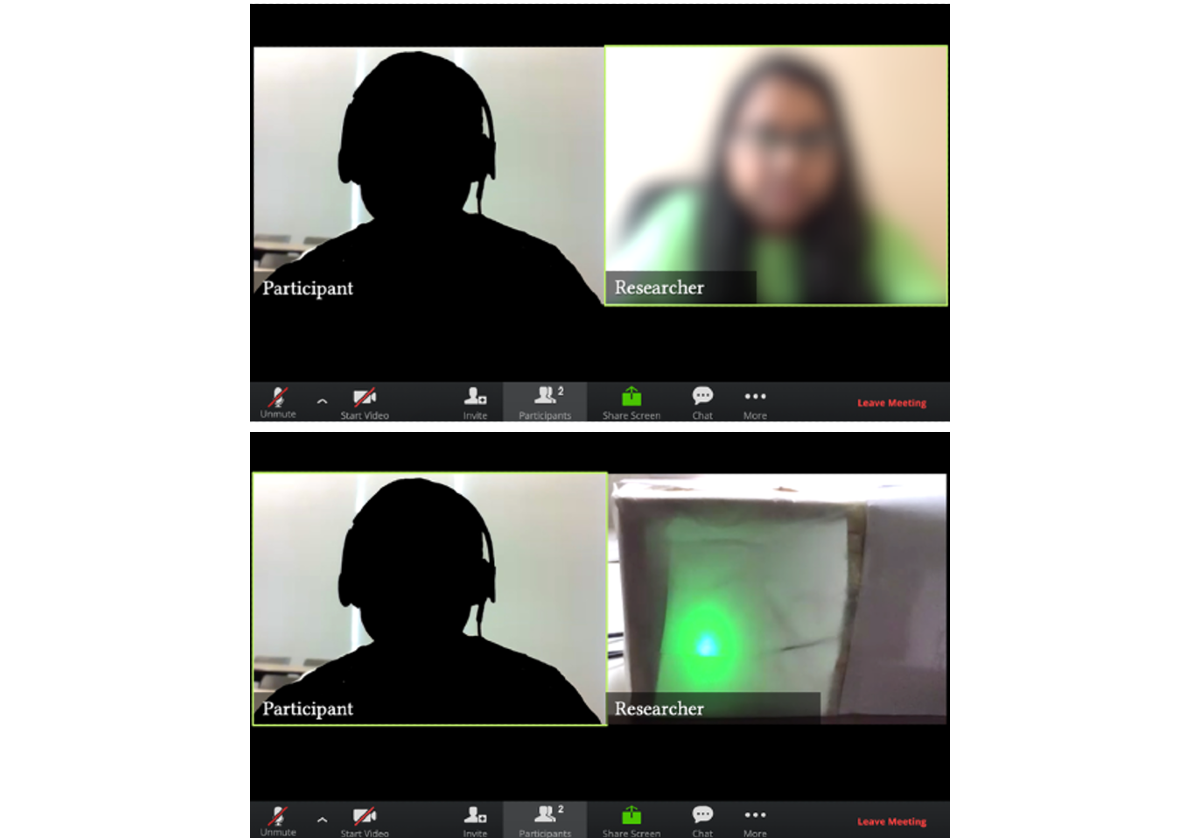
The study setup for the videoconferencing-based user study. In each video conferencing session, the participant and the researcher logged in with their cameras turned on. The researcher started the videoconferencing session by showing themselves in the video (top image) and then showed the Motivational Interviewing Conversational Agent (MICA) prototype (bottom image) on the screen only when the user study began. The researcher then turned the camera back to them (top image) for the end of the study interview.

As MICA is designed to be used repeatedly over time, rather than only once, we designed the study to test potential changes in participants’ experiences in using the MICA for the second time. Owing to the COVID-19 pandemic [72], we conducted the study on the web via the Zoom videoconferencing service [73]. Each participant was asked to participate in 2 sessions via Zoom software [73], with a 2-week gap between the sessions (standard for MI sessions); however, the second session was optional. Participants were recruited through a screening survey posted on Philadelphia Craigslist [74]. In each session, beginning with an informed consent process, the participants filled out a pretest questionnaire containing questions on demographic information. The participants then started their interaction with MICA prototype, a 20-minute MI session starting with barrier identification to healthy eating and ending with goal-setting. The participants interacted directly with the MICA prototype through Zoom. After participants’ interaction with MICA, the researcher interviewed them about the points in their interaction and communication with MICA they would like to see changed and how they envision their children using MICA. The interview also consisted of questions on how often they would be willing to use MICA and 5-point Likert scales for the perceived usefulness of MICA in reflecting on previous eating habits and motivating them to change unhealthy eating habits. This Likert scale was adapted from the System Usability Scale [75], which uses a 5-point scale for capturing sentiments about a product and is a standard benchmarking scale used in user experience studies. This process was repeated in the second session to analyze whether the experiences and perceptions changed when they interacted with MICA for the second time. This way, we would test user experiences of the system beyond participants’ first impressions of the system, which is more common in user tests of CA in their early phases. The MICA testing session and the interview on Zoom were screen recorded and transcribed. A US $25 Amazon gift card was given to each participant as compensation for participating in each study session. Figure 3 shows a participant interacting with the researcher and the MICA prototype during the study.

### Participants

Our study was motivated by understanding the perceived usefulness and acceptability of MICA by parents serving as proxies for their children in healthy eating. Accordingly, we only conducted a study with the parents for this phase of the project. Our selection criteria included parents aged >18 years living in the Philadelphia area who were overweight or obese with a BMI [76] ≥25 kg/m^2^, had at least one child aged <18 years, were not cognitively impaired, could read and write English, and were interested in eating healthier together with the child (answered “yes” for the question “Are you interested in participating in a program that addresses healthy eating behavior with your child?”). We chose parents’ overweight or obese status as an inclusion criterion because it is a contributing factor to children’s overweight or obesity [77]. By targeting overweight or obese parents as proxies, MICA can further impact childhood obesity.

Of the 24 parents who signed up for the first session, 20 (83%) participated in the second session. The average age of the 24 parents was 41.08 (SD 10.53) years, of which 15 (63%) were female and 9 (37%) were male. The average BMI of the participants was 31.13 (SD 5.76) kg/m^2^. Each participant also provided information about their child aged <18 years and with whom the parent participant wanted to eat healthier together as a family. Table 1 shows the demographics of the parent participants.

**Table 1:** Participants’ demographics.

ID	Age (years)	Sex	Race	BMI (kg/m^2^)	Marital status	Child’s sex	Child’s age (years)	Present in session 2
P1	34	Female	Black or African American	25.1	Never married	Female	4	No
P2	37	Male	Asian	25.7	Divorced	Female	6	Yes
P3	35	Female	Other	42.1	Engaged	Female	6	Yes
P4	33	Male	White	27	Married	Female	5 months	Yes
P5	33	Female	White	31.9	Married	Female	7	No
P6	46	Female	White	28.3	Engaged	Male	8	Yes
P7	56	Female	White	45.2	Widowed	Male	11	Yes
P8	52	Female	White	35.9	Married	Male	17	Yes
P9	50	Female	Black or African American	27.8	Married	Female	16	Yes
P10	51	Female	White	27.2	Married	Female	8	Yes
P11	34	Female	White	26.5	Legally separated	Female	3	Yes
P12	47	Male	White	34.5	Married	Male	11	Yes
P13	45	Female	White	26.6	Married	Female	14	Yes
P14	48	Female	White	25	Married	Female	11	Yes
P15	27	Male	White	29	Never married	Male	5	No
P16	34	Male	White	34.6	Married	Female	9	Yes
P17	37	Female	Black or African American	26.6	Married	Male	5	Yes
P18	27	Female	White	36.3	Divorced	Male	2	Yes
P19	70	Male	White	30.1	Married	Male	12	Yes
P20	37	Male	White	29	Married	Male	5	Yes
P21	50	Female	White	36.7	Divorced	Male	10	Yes
P22	29	Male	White	25.8	Never married	Female	6	Yes
P23	32	Male	Asian	30.1	Married	Female	7	Yes
P24	42	Female	Black or African American	40.2	Never married	Male	17	No

### Data Analysis

We transcribed the interviews and conducted a thematic analysis based on the grounded theory by Strauss and Corbin [78]. We used a constructivist framework to identify the participants’ perceptions of MICA and their suggestions for improving it to help them change their own and their children’s eating behaviors. The first author conducted an initial open coding on randomly selected 4 interview transcripts (approximately 10% of the total transcripts), generated codes, and presented them to the project team. On agreement and discussion from the team, the first author then continued to analyze the 40 transcripts from the 2 sessions based on the agreed codes. The analysis was done using NVivo 12 [79]. The research team met weekly to discuss newly emerged codes and themes to maintain agreement as a team. The coding structure was refined throughout the iterative cycles of discussions. After completion of the coding process, the team conducted affinity mapping [80] to identify broader themes. The team also analyzed the results of the questions on 5-point Likert scales and frequencies using descriptive statistics. As discussed in the Findings section, these measures provide a quantitative assessment of MICA’s perceived usefulness between sessions 1 and 2.

### Ethics Approval

This study was approved by Drexel University’s Institutional Review Board (1906007221-A004).

## Results

### Overview

From the interviews gathered in both sessions, the participants shared how a MICA could help them to self-reflect and gain motivation for eating healthy and, as a result, be a positive influence on their children. Participants shared ways in which MICA can support them to help their children eat healthy while ensuring safety as a priority and being mindful of the complex social dynamics around children’s healthy eating behaviors. The participants also shared how MICA could improve interactions to be more effective and usable.

### MICA Supporting the Proxy Role of Parents

#### Overview

The participants felt MICA could help them self-reflect on their previous habits to make changes to their eating habits. This perception remained consistent between the 2 sessions. The participants also perceived MICA as motivational, as it helped them feel supported and motivated their eating goals amid their parenting burden. MICA could then help parents positively influence their children’s eating behaviors.

#### MICA as an External Support Could Help Parents Self-reflect and Gain Motivation in Playing a Proxy Role

All participants reported being either familiar with or having experience in interacting with a CA, such as Google Home, Amazon Alexa, Samsung Bixby, or Apple Siri. For the participants, the difference between MICA and other CAs was that MICA asked healthy eating–related questions that made them reflect on their previous eating habits (P4, P14, P16, and P21). This self-reflection could help them influence their children’s eating behaviors as a proxy. The participants also saw MICA as a “close friend that motivated and nudged [them] to eat healthier” (P8, P11, P16, P19, and P23). When asked to choose 1 eating problem they wanted to solve as a parent who may be role modeling the eating habits of their children, participants chose eating junk food (5/24, 21%), overeating (4/24, 17%), emotional overeating (3/24, 13%), nighttime snacking (4/24, 17%), eating too much sugar (3/24, 13%), stress eating (2/24, 8%), eating fast food a lot (2/24, 8%), and the ability to self-control their eating (1/24, 4%).

As the participants would often get busy taking care of their children and helping them eat healthy, having external support in MICA was perceived as helpful. MICA could help participants understand where they were going wrong with their eating habits and what needed to be changed. By using MI in its script, MICA was able to gauge deeper into how the participants’ eating habits related to the family’s eating habits. The MI script allowed the participants to understand the current state of the family’s eating habits before thinking about how to change their own eating habits. Phrases such as “I see, tell me more” helped participants to reflect deep into the response they gave to MICA:

I like that if she [MICA] asked me a question and I did answer–It was as if it [MICA] understood my response and it [MICA] went back and it [MICA] would ask me another follow-up question based off of that answer.

P15

But I remember when I was talking about the energy–I kept my answer very short, and then the follow-up question was, “Can you please tell me a bit more about that.” So, it shows that she [MICA] will go the extra mile.

P11

Following up on a question made the participants (7/24, 29%) feel heard and cared about, a need that often gets ignored for parents serving as a proxy for their children. Overall, 42% (10/24) of participants especially liked the question, “If you were to stick with your current plan to change, where do you see yourself in 5 years?” as it made them think about the long-term effects of changing their family’s eating habits. This question also helped the participants prioritize activities for themselves and their children that could lead to a long-term behavior change. In all, 38% (9/24) of participants also expressed that the process of conversing with MICA made them verbalize their actions and kept them “accountable” for their eating goals. Being accountable to achieve their goals becomes essential for parents serving as a proxy for their children who want to bring about the same change in their children.

On the basis of participants’ perception of MICA as a tool for self-reflection, we followed up with a Likert-scale question to gauge the effectiveness of MICA. For the question of *whether MICA helps to reflect on previous eating habits* on a 5-point Likert scale of 0 being “not helpful” and 5 being “very helpful,” the median score was 4 (IQR 3-5) in session 1, leaning toward helpful or very helpful. For session 2, the median score was 4 (IQR 4-4). The participants explained this perception by sharing that verbally conversing with MICA pushed them to think more about their previous eating decisions:

[MICA] just kind of helps bring to light what’s been happening forever, and just kind of realizing what I was doing was a very helpful thing, you know. If I am not made aware of things, then I probably won’t stick to my goals. You know, I just kind of fell into a slump, and so reacting to MICA kind of helps realize what I was doing. So, it was very helpful.

P4

Furthermore, 50% (12/24) of participants perceived MICA as “motivational” because of the positive reinforcement (9/24, 38%) and affirmations (6/24, 25%). The MICA being “motivational” could help participants adhere to their healthy eating goals, especially when their self-motivation was low. This problem frequently happened during their caregiving process as a proxy, where parents often felt that they had no other support:

But having somebody speak to, motivational, you know, I guess it motivated me to want to do this. I can do this.

P8

It would leave me feeling like, “Okay, I think I got this. MICA thinks I can do this. I think I can do this, even if nobody else in my life thinks I can,” you know, there is something backing me up.

P16

On the basis of the participants’ perception of MICA as motivational, we followed up with a Likert-scale question. For the scale measuring *whether MICA motivated participants to change eating habits*, the median scores were 4 (IQR 3-5) and 4 (IQR 4-5) for sessions 1 and 2, respectively. MICA’s MI script used empathetic phrases such as “I can understand how difficult it can be to bring about a change such as this” to empathize with participants’ problems as proxies and motivate them to overcome those problems:

But it doesn’t sound as though [MICA] is just reading a script. It’s like there was a lot more thought put into the process, and I will definitely say a lot more empathy also. It’s very much appreciated because it goes to show that even though [MICA] is, you know, not a human being that they [MICA] can still feel for you and your struggle.

P3

The results indicated the acceptability of MICA as a tool for helping the participants reflect on their eating habits that affect their children, despite their busy lives as parents serving as proxies. MICA was also perceived to provide mental support to the participants and motivate them to eat healthy. As MICA helps participants to eat healthy, they will be better motivated and supported to bring about the same behavior change in their children.

#### MICA Could Help Parents to Positively Influence Their Children as a Proxy

Currently, MICA focuses on helping parents change their own eating habits. As a proxy for behavior change, parents are then motivated to bring about the same behavior change in their children. Participants shared that MICA could help them replicate their healthy eating habits in their children. To better achieve this goal, the participants wanted MICA to provide them with informational support so that they could independently form strategies with their children for eating healthy.

A challenge that 29% (7/24) of participants faced in their everyday lives was that although they encouraged their children to eat healthy, the participants themselves did not eat healthy. Being their children’s primary caregivers and having trouble adopting healthy eating habits of their own, the participants found it challenging to incorporate healthy food items in their children’s diets. This problem inhibited the parents from becoming good role models or proxies for their children to help them adopt healthy eating habits:

I [parent] am suffering to get her [child] to eat the greens and veggies or whatever. But if I don’t eat it, then who am I to force something on her if I don’t make it for myself or like it. So, it has been a challenge.

P11

To address this problem, the participants perceived that incorporating healthy eating habits themselves with the help of MICA would motivate their children to eat healthy with them:

Him watching me interact with [MICA] and eating better, he [child] may, you know, want to do the same thing.

P20

Well, I feel like if I’m eating healthier, and I am pretty much the main cook in the house. Then I’m going to incorporate healthier items into his [child’s] plate.

P15

MICA’s current design following the MI principles probes participants to develop healthy eating strategies of their own. For young children to best respond to these MI strategies through their parents, the participants wanted to be involved in their children’s food decisions, with the aim of their children gaining greater control over their food decisions gradually over time. They wanted MICA to offer suggestions on healthy eating, such as the food items they could incorporate as part of healthy eating (P7, P15, P21, and P23). Nutritional information about the different food items was another suggestion from MICA that 33% (8/24) of participants wanted to make their children aware of the food items they consumed. Apart from the motivation that MICA provides, the participants believed that these suggestions and information would help them prepare their children to make informed food choices over time and gain more autonomy over their eating habits:

And, you know, [parent] can help them obtain really good habits when they’re young. I [parent] used to have really good habits because my mom would teach me about the kind of the food groups and how those things react. It’s those changes in my adult life that have gotten me to where I am. So, I would just think would be a great way for kids to get more information and make better choices for themselves.

P7

Parenting, however, is often not an easy task to be undertaken by 1 guardian. Multiple family members and households are often involved in influencing children’s behavior.

### MICA Safely Working With Complex Family Dynamics and Trust

#### Overview

One of the challenges participants wanted to resolve about their children’s eating habits was managing conflicting expectations between parents, guardians, and family members. They felt MICA could work as third-party support to help them navigate this complex dynamic. Furthermore, participants wanted MICA’s conversational approach to address their concerns around existing health behavior change tools by putting safety before behavior change goals.

#### MICA Should Understand and Work With Complex Family Dynamics as Third-Party Support

Participants felt MICA could help them keep track of their children’s eating habits when dealing with complex family dynamics, such as managing children between multiple households and differing parenting styles among guardians. Participants felt it would be useful if MICA plays a third-party role, such as providing them suggestions and reminders to help their children understand the importance of eating healthy.

P16, P21, and P22 were concerned that their children were developing unhealthy eating habits or gaining a negative influence when visiting the other parent from whom they were separated. This finding also portrayed the complex social dynamics involved in families’ healthy eating:

Our nine-year-old talked to my wife that she was feeling fat. And we share custody with my ex, and my ex had an eating disorder. My ex’s mother actually tells my daughter that she’s too fat.

P16

Similarly, P3 felt that she did not have the proper authority to teach her partner’s child to eat healthier. Such social dynamics involved in families’ diverse social relationship settings make it difficult for participants to take complete control of healthy eating practices. Some participants (9/24, 38%) were also single parents or separated from their partners. In such cases, MICA could become a “support system” to help the participants keep track of the child’s eating habits and provide a consistent eating regime for the child even while visiting the other parent:

[MICA] provid[ing] affirmations or reminders to the other parent is super helpful because when she’s [child] away from us, she [child] doesn’t have a huge social support system to combat those negative influences. So, for her, even if MICA just says to the other parent, “Hey, remember to eat veggies and make sure to get enough protein and starches,” then, in that case, MICA is providing a great influence that she [child] otherwise wouldn’t get, um, other than that.

P16

Some participants (7/24, 29%) also expressed difficulty conveying the importance of eating healthy to their children:

I still find it difficult for me to explain to my child about the benefits of healthy eating.

P23

The participants (7/24, 29%) had a tough time making their children “listen” to them, as their children ignored their suggestions for eating healthy food that they did not like to eat:

99% of the time they [children] don’t listen. They’re [children] not open to trying new things.

P20

To help with this challenge, P13, P14, P16, P14, and P22 envisioned using MICA to remind their children of the benefits and ways of eating healthy. P22 suggested having MICA help them to “remind [child] to eat vegetables and giving [child] information and why it’s important to eat vegetables and healthy food.”

#### MICA Should Foster Trust and Safety by “Figuring Things Out Together” and Involving Experts

The participants were concerned that pushing children to eat healthier can potentially generate side effects such as undereating. P4, P16, and P19 saw undereating because of such peer pressure as a challenge that MICA could potentially address; they thought MICA could approach healthy eating differently than other interventions in ways parents and children can talk about it together and “figure out things”:

I would hope that she [child] could ask questions, like, “How much should I’ve eaten” or “What did I eat.” Or, not make [eating healthy] turn into a thing to where she’s so focused that she developed an eating disorder. But, something that’s more like a tool [MICA] where, you know, we can talk about eating together and figure out different things.

P4

The participants felt that MICA could support them to help their children in the longer run by preparing them to make informed decisions about their eating habits. However, the participants did not completely trust MICA’s suggestions for helping them with their children. Especially when the children were younger, they were concerned about the misuse of CAs to aid in undereating or overeating (P11, P12, P18, and P19). P16 and P19 also desired the involvement of a health care provider or at least some parental supervision over how MICA incorporates suggestions for their children because they did not trust that MICA could handle sensitive complexities around pressure to eat healthier, potentially leading to undereating:

I don’t know that without like a physician involved, I wouldn’t really want to use it for anything more than just generic nutrition advice. Like, what’s healthy about an apple– I would completely trust MICA to tell me that. But, if my daughter decided to come up with a weight loss plan and talked to me about it, I would be really, really concerned about the ability of a voice assistant to help me handle the complexities of that.

P16

Here, we discovered that the participants wanted MICA to help them guide their children on healthy eating. However, enabling CAs as a guide for helping parents with their children can be harmful to children, as CAs possess the risk of being misused by external parties without parents’ knowledge. CAs can then generate inaccurate advice and suggestions, resulting in more harm than good. Parents may not be informed of such harmful interactions, making it difficult to keep track of the harm children may be going through.

### Enhancing the Interaction Experience With MICA

#### Overview

The participants perceived that MICA’s motivational and empathetic responses could make them feel supported in their parenting process. MICA was then compared with a therapist or a nutritionist because of the accountability and empathy it provided. Because of this perception, participants shared how MICA’s interaction can be improved for a better therapeutic experience. One of the major themes that emerged was whether to accommodate human-like interactions over non–human-like interactions. Participants also wanted MICA to enhance goal-relevant interactions in its MI scripts. Finally, how often, where, and how long they wanted to interact with MICA also mattered to the participants.

#### MICA Should Customize Human-Like Versus Non–Human-Like Interactions to Parents’ Preferences

A recurring theme in improving MICA was to make it more or less human-like. Being more human would involve making MICA’s conversations to be more “natural” (7/24, 29%) and the voice to be less “robotic” (15/24, 63%). MICA’s human-like nature would help participants feel less lonely, serving as a proxy for behavior change in their children. On the other hand, some participants (8/24, 33%) preferred that MICA was “artificial” so that they could talk about their and their children’s eating problems in a “safe manner to an AI without being judged.”

The participants often used human pronouns to refer to MICA, such as she or he. They felt that MICA ingrained components of a human-like conversation, as it acknowledged their previous responses, indicating that MICA was following up on their responses. This human-like nature of MICA could help the participants feel supported and less lonely in their behavior change journey along with their day-to-day caregiving tasks. MICA also referred to previous answers given by the participants both during a session and between the first and second sessions. The participants liked this call back to their previous conversations, as it meant to them that there was somebody (MICA) to check up on them as well, a task which they did daily for their children:

I like that it [MICA] was able to recognize what you’re saying and then be able to provide thoughtful follow-up questions for it.

P2

She [MICA] remembered what I did when I said my problem was last week, and now she’s checking on me to see how I’m doing with that. How nice obtaining that information!

P14

For improving the human-like interaction further, the participants wanted longer, more drawn-out conversations that look into “the reasons behind an answer” (P12, P14, P15, and P22) for behavior change, as people often try to get to the bottom of an answer in human-human conversations:

To give it more of a feel like I’m actually talking to a person– have conversations where I can expand on a thought and get a little bit more detailed– where I feel like she’s [MICA] going to understand me.

P14

I would say probably going more in-depth to each aspect. So, it [MICA] kind of digs into where you’re at, what your plans are, and things like that.

P15

On achieving a goal the participants had set for themselves, MICA responded with positive phrases such as “That’s awesome!” or “I am glad to hear that.” The participants perceived such interactions could motivate them to continue with their goals and offer mental support that is hard to receive as parents serving as a proxy. However, P9 found it “awkward” to hear words of encouragement from an artificial device. She preferred more direct responses from MICA, wherein no affirmations or positive words such as “That is great to hear” or “Awesome!” were used. P21 also found positive encouragement from MICA “not as sincere as a human” as she perceived it not to be grounded in “emotional reality.” In contrast to making MICA human-like, P7, P12, P18, P20, and P21 preferred MICA not to be human-like, as it meant it would not “judge” them or “get mad”:

I mean, honestly, it’s probably easier for me to answer a question from an AI about my eating habits or what I think I’m doing wrong or something like that than it would be to answer questions like that from a real person. Because I don’t feel like the AI is going to judge.

P21

MICA not being human-like made it “trustworthy” (P11 and P23) and “comfortable for sharing things like weight” (P18) for some participants in the context of accomplishing their eating goals, especially information related to their children.

It lowers that apprehension that you have with things or people or whatever and immediately ups the trust that you have with them.

P11

Participants especially preferred MICA being non–human-like in the context of adopting healthier eating habits, which can become a sensitive or personal topic to share with a human-like partner.

Participants liked that MICA followed up on their responses and suggested ways to make it more human-like. On the other hand, 29% (7/24) of participants preferred MICA to be non–human-like. This finding calls for giving participants the option to change MICA according to their preferences, making them feel supported in behavior change while providing sufficient help as a proxy for their children to eat healthier.

#### MICA’s MI Script Should Reflect Customized Goal-Relevant Questions and Suggestions for Parents

MICA helping participants achieve their own goals for eating healthy would help them replicate the same behavior change in their children. In this regard, 38% (9/24) of participants shared the kinds of questions they prefer MICA ask to help them make better decisions for themselves and their children. The questions asked by MICA as part of its MI script were thought to be relevant and “thought provoking.”

Some participants (10/24, 42%) enjoyed the time-based question as part of the goal-setting agenda, wherein MICA asked them if they stuck to their goals, where they would see themselves in 5 years. This form of time-based goal setting could help put things into perspective and provide the necessary motivation for participants to change their and their children’s eating habits. However, P5, P14, P15, and P17 also wanted MICA to change the period of these questions to shorter durations of a week, a month, or a year. They perceived that being able to change this period could provide them the necessary motivation to achieve short-term goals:

And, maybe, set a weekly goal amount. As the weeks go by, you know, with weight loss, in the beginning, you lose a more significant amount, and then after that it starts to go a pound here and a pound there, you know. So, maybe that could also be taken into consideration as time goes on. Because, the way she [MICA] asked the question, I kind of felt it’s long-term because she said that about five years from now.

P14

As parents shape their children’s eating behaviors from an early age, having questions that reflect the problems and needs of parents would be essential for supporting the participants as proxies. For improving MICA to be reflective of their goals and needs, 38% (9/24) of participants wanted MICA to ask relevant questions first to gauge their daily dietary behaviors:

It didn’t get specific as to the foods I’m eating. Do I eat a lot of salty snacks? Am I more loading up on carbs? It [MICA] really didn’t ask me all those kinds of questions that much.

P13

Similarly, P4 desired more questions on his problem of nighttime snacking, asking about what food items he consumed late at night and what he would do to overcome this problem. P15 and P22 wanted MICA to ask about the food items they liked to eat and “couldn’t resist.” P9, P10, P11, P20, and P22 wanted more questions on what food items they were consuming every day and what they would and would not like to eat as a part of healthy eating. The participants then wanted MICA to adapt to their individual goals and needs by asking them questions about their plans for accomplishing their goals:

[MICA] can ask questions such as “What are you planning on having today for breakfast?” or just maybe like mix it up every once in a while, and say, “Hey, what’s your plan for today with your eating goals?” Or, maybe, suggest to me, “Hey, have you ridden your bike today?” or, “Have you gone for a walk today?” [MICA] gives helpful little tips about keeping healthy and staying healthy.

P10

Questions will be centered around like “How are you doing with this?” or “What have you eaten today?” and those types of questions, I think, could enhance the personal aspect of the interaction with [MICA].

P20

This interaction based on the participants’ dietary choices and problems could help them make informed decisions about which food items to eat and which to avoid, helping to replicate the same for their children. Another way some participants (7/24, 29%) wanted to improve their interaction with MICA was by MICA making its suggestions specific to their goals. P8 wanted to be able to decide what to eat for a meal based on what MICA marked as healthy, for instance. P23 suggested MICA inquire about the “types of food consumed” during a day to recommend coping strategies; P15 asked for “sharing information about like one soda has this many calories”; and P10 asked for MICA to keep track of the sugar-based foods consumed during the day given their prediabetic condition. Such information provided by MICA would help participants take better care of themselves and, in turn, their children.

#### Location, Frequency, and Duration of Interacting With MICA Matters

When we probed the participants on how they might envision using MICA in their home contexts, the initial reaction was where they would place it. The meanings that the participants associated with the physical space about healthy eating and how MICA could best support them in their daily caregiving tasks mattered in their perceived choice of the physical placement of MICA. The participants wanted to place MICA in the kitchen (16/24, 67%), bedroom (6/24, 25%), living room (1/24, 4%), or working desk (1/24, 4%). This perception remained consistent between the 2 sessions. The kitchen was the place in the house where the participants made most of their healthy eating decisions together with their children. Participants shared that having MICA there as a reminder for healthy eating could help the participants not make “unhealthy” decisions for themselves as well as their children and could keep them accountable:

[Kitchen] is where the offending actions have been. So, if she [MICA] was there [in the kitchen] as a friendly reminder, you know, you’ve got goals so be accountable to them.

P7

So, you know, if it’s in another room versus the culprit room we’ll call it where most of the unhealthy eating happens, which is like the kitchen area. If MICA is there [in the kitchen], I’ll be more inclined to have her perked up by, you know, addressing her and the conversation will start.

P3

The perceived frequency with which the participants saw themselves conversing with MICA decreased from sessions 1 to 2, as they realized the feasibility of taking time out from their busy caregiving schedules to converse with MICA. For session 1, of the 24 participants, 18 (75%) participants reported wanting to interact with MICA daily, 5 (21%) participants every week, and 1 (4%) participant did not report. For session 2, of the 20 participants who followed up, 9 (38%) participants wanted daily interaction, 1 (4%) wanted interaction with the MICA every 3 to 4 days, 6 (25%) wanted it to be weekly, 2 (8%) preferred biweekly, 1 (4%) participant reported monthly interaction, and 1 (4%) occasionally when desired. P16, P19, P20, P23, P4, and P8 wanted daily interactions with MICA, as it meant receiving motivation to adhere to their goals regularly. After the second session, P2, P3, P18, and P21 felt that less frequent interactions would give them more time to help their children while making sufficient progress toward their goals, which they could then report back to MICA. P21 elaborated on her preferred frequency of a week as follows:

Every day would be too much. But over the course of a week, you’ve had highs and you’ve had lows. And so, I think that would be a better self-reflective period.

P21

P4, P13, P21, P23, and P24 also said they would prefer less frequent interactions with MICA as they often got busy providing care and would find it challenging to take time out to converse with MICA. Participants (9/24, 38%) with similar opinions on frequency still wanted a “daily reminder system,” which would softly nudge them toward their goal:

So, I would want [MICA] to do the reminders every day. Not in an accusatory way, but like, “Hey, we’re able to go take your walk today.” And that really might make me be like, yeah, I need to go do that.

P8

The participants (9/24, 38%) also felt they often did not have enough time to commit to a uniform session length with MICA. As the participants would get busy taking care of their children while trying to change their own and their children’s behavior, the conversation length could vary depending on the time the participants had for their interactive sessions with MICA. The participants perceived that having the current MICA session followed by smaller sessions based on the participants’ availability would be feasible for the participants:

I’m wondering if you had many sessions with MICA–this session is an introductory session and then there are some follow-ups, you know, just checking in or that kind of thing.

P7

This change in perception for how often and how long they want to interact with MICA after the second session highlights the need for MICA to cater to the changing needs and preferences of parents serving as a proxy for their children. Participants only knew what felt right after having experienced it. Using MICA for the second time in 2 weeks made them realize they needed more time to achieve the goal they had stated they wanted to achieve during the first session. For parents serving as a proxy, whose needs and goals mutually affect the needs and goals of their children, MICA should give parents the option to change the frequency and length of the sessions as desired by the parent.

The results report on MICA’s perceived usefulness in helping participants reflect on their eating habits and stay motivated toward their healthy eating goals, which indirectly helps their children to adopt healthy eating habits. Participants perceived that it mattered where MICA is placed or how often they interact with MICA and for how long. These configurations would then be adjusted based on each participant’s family’s unique goals and needs around accountability and motivation.

The results show the different roles and functions of an MI-based CA that can help parents eat healthy with their children by serving as proxies. The findings point toward the design of CAs that go beyond supporting a target individual of behavior change and supporting behaviors that require coordination between multiple persons, such as between parents and their children.

## Discussion

### Overview

Our research was primarily motivated by the lack of accessible technological support for parents, who often act as proxies for their children for behavior change. We conducted a study to test user experience around the concept of an MI-based CA, MICA, that supports parents to help them eat healthier together with their children. We learned several lessons, starting with requirements for supporting behavior change that requires coordination between parents and children in various social complexities. We learned how MICA’s interactions could be customized to cater to the goals and needs of parents because they serve as proxies for their children’s healthy eating goals. We also learned how MI-based conversations can be incorporated in CAs to cater to the needs and goals of parents serving as proxies. We end with discussing broader implications for designing MI-based CAs for those serving as proxies for health behavior change.

### Principal Findings

#### On MI-Based CAs Supporting Parents to Safely Manage Children’s Behaviors in the Context of Healthy Eating

Existing research suggests using CAs to track children’s food intake and then nudging the children to eat healthier under parents’ rules [2]. The literature also indicates parents’ apprehension about their children interacting directly with a CA [2,41]. Currently, MICA implicitly helps parents serve as proxies for their children in eating healthy. MICA provides support to parents to change their eating behaviors, who can then motivate their children to eat healthy. Parent participants in our study suggested acting as role models for their children, wherein their children could replicate their eating habits or learn from them as they made progress with CAs. This proxy relationship can be beneficial for both parents and children, as they motivate each other to eat better [81]. In addition to implicit support, our findings show that parents wanted MICA to provide explicit support and information for help with their children. For example, MICA can suggest strategies to parents around conversations with their children about their eating habits and come up with goals together as a unit.

Diverse family structures (eg, parenting partner’s child, one’s child spending time at another parent’s household) in our participant pool generated complex social dynamics that the participants thought MICA should address. Studies have discussed CAs serving as social actors to mediate communication between family members [31,82,83]. From our findings, MICA could help parents provide consistency in the changing lives of their children for maintaining a behavior change. This consistency could be brought about by MICA serving as a uniform guide for the child toward making healthy food decisions, even when they were with the other parent who might have different approaches to healthy eating. Thus, CAs can help parents change their children’s behavior even in their temporary absence.

The participants expressed that MICA’s MI-based approach can contribute to strategies around helping parents support children to gain autonomy over their eating decisions from an early age. Although a parent may be comfortable involving a CA for guidance, CAs always have the potential of being misused. As parents develop trust in the CA through continued use, external parties can use the CA to manipulate the information given to the parents and therefore to the children. Moreover, language models are often not competent enough to interact in a safe and factually correct language consistently [84]. To find a balance between child safety and CAs supporting parenting, parents should be given appropriate background knowledge about CAs’ suggestions and recommendations for healthy eating strategies. Such transparency from CA designers about the trustworthiness of such technologies is a good first step toward developing CAs for parenting [85]. In this regard, regulating the suggestions given by MICA to parents acting as proxies for their children becomes an essential feature. Involving a health care provider or an MI expert to regulate MICA’s interaction would be helpful, similar to how the current MICA is designed. Every response of MICA is strictly designed by an MI expert. This finding highlights the need to make it explicit to parents that MICA is strictly guided by an MI or health expert; explicitly stating this information to parents can increase trust among them for similar CAs.

Undereating or overeating due to peer pressure was a prevalent problem among the children of the participants in our study. To overcome this problem in their children, participants suggested MICA help them in monitoring inaccurate advice that their children may receive through other external channels, such as peers or social media [86]. Therefore, in addition to ensuring that the content’s credibility is high, finding ways to support parents to moderate CAs’ content will be helpful. However, given the largely black-boxed algorithms of artificial intelligence–based CAs, moderating CA’s responses from parents’ perspectives will be a critical design challenge.

#### On Customizing Interactions of MI-Based CAs for Parents as Proxies

Customized conversational strategies in CAs improve system performance, usability, and efficiency [87-89]. Parents, who are the informal caregivers of their children, often have the responsibility of making informed decisions on behalf of the children. Findings from the participants supported the need to include customizable features to meet the varying needs of parents serving as proxies. For example, the parent participants wanted to change their conversation length with MICA depending on how much time they had and their personal preferences. Participants thought that the current MICA session length followed by smaller sessions based on their availability would be feasible for them. Parents acting as proxies for the persons they care for are often overburdened physically and mentally [23]. Therefore, it becomes necessary for these proxies to adjust certain CA features depending on their availability and use. Studies have also shown that adapting conversation length based on user preferences may lead to fewer concerns about content credibility and may increase trust in the system [90]. Other standard features that CAs could adapt to proxies’ preferences are the agent’s verbiage, sex, accent, language, and volume.

The meaning that the participants associated with the physical space for healthy eating affected the physical placement of MICA. MICA’s placement can be customized to the needs of the participants and how MICA can best support them in their daily caregiving tasks. A total of 67% (16/24) of participants wanted to place MICA in the kitchen, as the kitchen was the place in the house where the participants made most of their healthy eating decisions together with their children. The frequency of interaction with MICA also differed between the participants, but they wanted less frequent interactions after the second session. After the second session, the participants felt that less frequent interactions would give them more time to help their children while making sufficient progress toward their goals, which they could then report back to MICA.

Owing to its empathetical conversational style, our findings showed that MICA can be seen as a human-like agent. Aligned with previous research on people’s perception of CAs [91-93], empathetic responses from a CA would make it appear more like a human, thus helping to alleviate the social isolation of a parent as a proxy to some extent. Parents may call a CA by the name or a human pronoun (eg, “her”) to keep them accountable and feel empathy and support from it. However, being human-like is not always perceived as favorable by parents [94], as it often can be perceived as insincere, coming from an artificial agent [95]. Empathetic and motivational responses from an artificial device can be “awkward” and “weird,” as was found by 33% (8/24) of participants in our study as well. Of those parent participants, 21% (5/24), even preferred that MICA was an artificial being—it appeared to “not judge” them on their eating behaviors and made them feel “comfortable” to share their weight. This contrasting perception of MICA in our findings on what is appropriate or effective calls for the human-like nature of CAs to be modified based on the context and parents’ preferences. The preference for how human-like a CA should be may change for the same parent at different times of the day, similar to how 4% (1/24) of participants preferred to hear motivational words at the start of the day compared with the rest of the day. This customizable human-like nature of CAs would make parents serving as proxies feel supported and help them focus on their caregiving tasks at hand.

#### On Designing MI-Based Conversation Scripts of CAs for Parents as Proxies

Parents help shape their children’s eating habits from an early age, and involving parents in interventions as core participants helps improve children’s healthy eating [7,20,21]. Accordingly, helping parents eat healthier would help their children eat healthier as well. Parents often find themselves overburdened with providing care, leaving them vulnerable to mental health problems and a lack of support. Through the use of MI, MICA could help the participants feel supported and motivated in their healthy eating journey. The MI technique emphasizes a personalized approach to behavior change, offering autonomy to parents over their behavioral goals and decisions. This form of self-regulation and positive motivation may result in the long-term maintenance of behavior change, especially for parents as proxies, who may require motivational support to bring about a behavior change in themselves and their children [96,97]. However, such MI technique requires a continuous conversational exchange. Parents serving as a proxy are continually asked to answer questions that would probe about their and their children’s hidden challenges, motivations, and goals.

By incorporating the MI technique, MICA allows parents as proxies to develop their own goals based on their context, strength, and needs. MICA probing participants about their barriers to eating healthy led them to self-reflect on their eating problems and solutions, which were all essentially generated by the participant, not by MICA. The participants felt that having an external member point out opportunities in their behavior change regime for self-reflection could greatly help them, as they did not have the time or the support to do so themselves. Such self-reflection could motivate the parents to bring about the same behavior change in their children for whom they are proxies. The work by Chen et al [3] on parents using voice-based technologies at mealtime found that parents did not like to receive instructions from a CA, as they feared it would make them and their families dependent on the CA. Our work further adds to this finding that even with a technology-mediated approach, using MI as a conversational tool for interacting with parents will give them autonomy and motivation toward their health decisions. This form of interaction can further help parents feel more confident in helping themselves and their children.

Asking time-based questions as part of the goal-setting phase of MI for eating healthy was also preferred by the participants to stay motivated. The time-based question “Where do you see yourself in 1 year?” could be asked for goals that they could accomplish within a short period; for example, not eating out frequently. On the other hand, “Where do you see yourself in 5 years?” could be asked for goals that required more time to accomplish according to the participants; for example, the goal of losing a specific amount of weight. Such time-based goal-setting can be a catalyst for providing the necessary motivation to bring about a behavior change in parents [98]. Parents can, in turn, help their children achieve the same behavior change. The period in which the parents see themselves accomplishing a goal is also dependent on how much time they can devote to the goal from their caregiving schedules.

MI-based conversations offer CAs the opportunity to dive deep into the parents’ problems, as is possible in human-human conversations. Furthermore, as MICA’s MI-based conversation with the participants was not restricted to performing a task, the participants felt the conversation to be more “natural” [99]. CAs are then compared with a “therapist” or a “guide/coach,” although more complex language models have to be developed for CAs to become truly conversational [84]. Although MICA helped participants reflect on their responses, 17% (4/24) of participants still felt that conversations with MICA could be more drawn out. MICA has the potential of going deep into the problems faced by parents and understanding where they are with a goal. Although diving deep into the parents’ problems and coming up with solutions is useful, changing the frequency of such conversations based on parents’ availability would increase retention of use, especially for parents who are otherwise occupied with providing care. Rather than conducting the same session every day, CAs should give daily reminders about the goals parents have already set for themselves and their children. Therefore, although MI can help parents as proxies gain confidence in their decisions, frequent MI conversations may not be deemed feasible. CAs should incorporate a balance between MI conversations and daily reminders, which must be customized according to the needs of the parents serving as proxies [90].

#### Toward CAs Supporting Those Serving as Proxies for Health Behavior Change

User testing parents’ experience of using MICA provided a building ground for designing CAs that cater to the needs of those serving as proxies beyond parents. Parents wanting to eat healthy together with their children is one example of a proxy relationship for health behavior change. In addition to parents supporting their children, proxy relationships can include any family member or friend supporting a close one. Such proxy relationships are common when the target individual is unable to directly interact with the source of behavior change [8,9]. Examples include adult children of older adults taking care of their parents physically and emotionally or a close friend helping their friend with multiple chronic conditions needing to exercise together for medical reasons. Another example is a spouse helping a cognitively declining spouse who needs to perform daily physical rehabilitation exercises. Current technology providing solutions for health behavior change poses a limitation of not going beyond the needs of individual users and accommodating the needs of such proxy relationships. Technological interventions may not be accessible to care receivers such as young children or individuals who lack access to technology because of socioeconomic reasons, including costs. Those who lack appropriate technical literacy, health literacy, or physical capacity to use such interventions directly are also at a disadvantage. Therefore, it is essential to design technologies that cater to such proxy relationships to address these gaps in fulfilling the unmet needs of individuals and their supporters. Little is known beyond how individuals or a group of individuals directly interact with health-focused CAs. Our study provides a starting point for the critical role CAs can play in leveraging social contexts, such as proxy, where multiple people are involved in an individual’s behavior change.

### Limitations and Future Work

This study was limited by the context of using MICA in a laboratory setting. Furthermore, as the study was conducted remotely due to the COVID-19 pandemic, the presence of a researcher during the interaction of the participants with MICA may have influenced their responses. Future studies with an improved MICA prototype could involve testing MICA with parents in the privacy of their households. The MICA prototype was tested in 2 sessions 2 weeks apart to understand changes in the participants’ perceptions and needs. To measure the long-term behavior change effects of MICA, a longitudinal study spanning weeks can be conducted in the future.

### Conclusions

As off-the-shelf CAs are increasingly adopted in people’s homes, opportunities to automate and increase access to behavior change techniques that benefit from conversational exchanges, such as MI, arise as a sustainable behavior change solution. Family, parents, and friends who act as a proxy for the behavior change of a target individual are often burdened with various support tasks. We developed an MI-based CA called MICA to help parents support their children better as a proxy for health behavior change. This study aimed to examine the acceptability and user experience around using MICA by parents serving as a proxy for their children in adopting healthy eating habits. From 44 user test sessions, several insights emerged around the design requirements of MI-based CAs that support multiple individuals involved in a person’s behavior change. Future work will examine how MICA can lead to effective long-term behavior change. This study is the first attempt at investigating how CAs can support behavior change influenced by multiple people. Our work contributes to the increased attention on CAs for health and how CAs can leverage such a critical social context—a proxy—in supporting the health of individuals who have limited ability to take care of themselves.

## Abbreviations

CA: conversational agent
MI: Motivational Interviewing
MICA: Motivational Interviewing Conversational Agent
TTM: transtheoretical model
